# Artificially designed hybrids facilitate efficient generation of high-resolution linkage maps

**DOI:** 10.1038/s41598-018-34431-6

**Published:** 2018-10-31

**Authors:** Kazutoshi Yoshitake, Yoji Igarashi, Misaki Mizukoshi, Shigeharu Kinoshita, Susumu Mitsuyama, Yutaka Suzuki, Kazuyoshi Saito, Shugo Watabe, Shuichi Asakawa

**Affiliations:** 1 Laboratory of Aquatic Molecular Biology and Biotechnology, Graduate School of Agricultural and Life Sciences, The University of Tokyo, 1-1-1 Yayoi, Bunkyo, Tokyo, 113-8657 Japan; 2 Department of Medical Genome Sciences, Graduate School of Frontier Sciences, The University of Tokyo, 5-1-5 Kashiwanoha, Kashiwa, Chiba, 277-8561 Japan; 3 Akita Prefectural Institute of Fisheries, Oga, Akita, 010-0531 Japan; 4 School of Marine Biosciences, Kitasato University, Sagamihara, Kanagawa 252-0373 Japan

## Abstract

When sequencing eukaryotic genomes, linkage maps are indispensable for building scaffolds to assemble and/or to validate chromosomes. However, current approaches to constructing linkage maps are limited by marker density and cost-effectiveness, especially for wild organisms. We have now devised a new strategy based on artificially generated hybrid organisms to acquire ultrahigh-density genomic markers at reduced cost and build highly accurate linkage maps. We have also developed the novel analysis pipeline Scaffold Extender with Low Depth Linkage Analysis (SELDLA) for data processing to generate linkage maps and draft genomes. Using SELDLA, linkage maps and improved genomes for two species of pufferfish, *Takifugu rubripes* and *Takifugu stictonotus*, were obtained simultaneously. The strategy is applicable to a wide range of sexually reproducing organisms, and could, therefore, accelerate the whole genome analysis of various organisms including fish, mollusks, amphibians, insects, plants, and even mammals.

## Introduction

New-generation technologies now enable whole-genome sequencing of any organism, although assembling long, high-quality genomes from short reads generated by these technologies remains challenging. Accordingly, methods such as Irys genome mapping (BioNano Genomics Inc.), Hi-C, Chicago library (Dovetail), and linked reads (10× Genomics) have been developed to generate longer scaffolds and improve assembly^1–4^. Nevertheless, genetic linkage analysis would likely remain essential to reconstruct most eukaryotic genomes, even if sequencing methods continue to advance. Indeed, linkage maps are used not only to assemble contigs/scaffolds, but also to compare and evaluate the assembled sequences.

Construction of conventional linkage maps based on microsatellite markers generally is costly and time-consuming, and the maps are of low resolution and genomic coverage due to the limited number of markers, usually several hundreds to several thousands^5^. Several new methods to construct linkage maps and assemble contigs/scaffolds have also been developed, including SNP array^6^, RAD-seq^7^, and genotyping-by-sequencing^8^. However, SNP arrays require SNP data and hundreds of arrays prepared in advance, and therefore may not be suitable for *de novo* genome analysis. In contrast tens of thousands of SNP markers are easily obtained by RAD-seq or genotyping-by-sequencing, although the distribution of such markers strongly depends on the restriction enzymes used. In addition, markers obtained from these methods are usually present at one per tens of kb; thus, they lie outside the length of most contigs and small scaffolds, and cannot be located on the genome.

**Figure 1:**
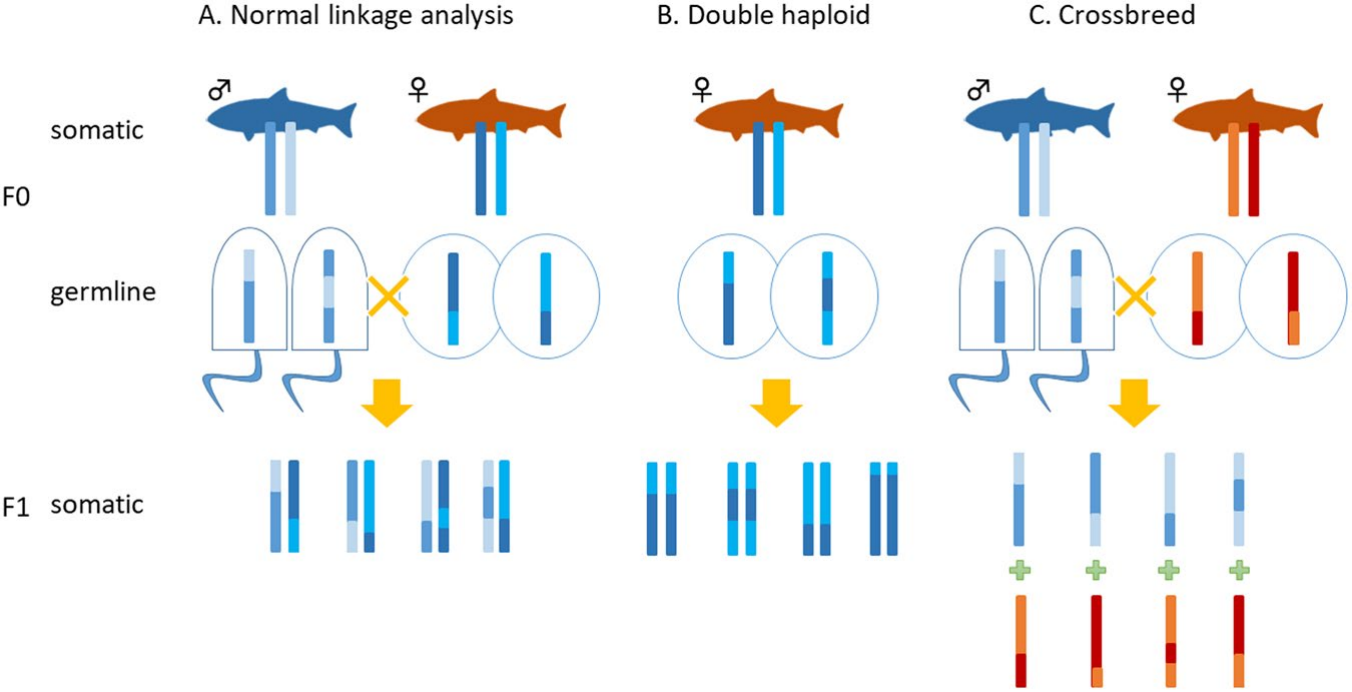
Comparison of linkage analysis methods. The types of individuals necessary for linkage analysis to extend genomic sequences are shown for each method. (**A**) Crosses and chromosomes for linkage analysis in normal wild populations. (**B**) Chromosomes of double haploid individuals. (**C**) Chromosomes of hybrid individuals.

Determining the two haplotypes in each individual is the main challenge in linkage analysis (Fig. 1A). Indeed, typing of polymorphic sites by genome sequencing generally requires coverage higher than 10-fold, on average, to distinguish between hetero- and homozygosity. As several hundred individuals are usually genotyped, a large volume of tasks would be required to achieve the required coverage for each individual. Such large tasks may thus prevent the use of whole-genome sequencing for this purpose, even if throughput from sequencing technologies significantly expands.

**Figure 2:**
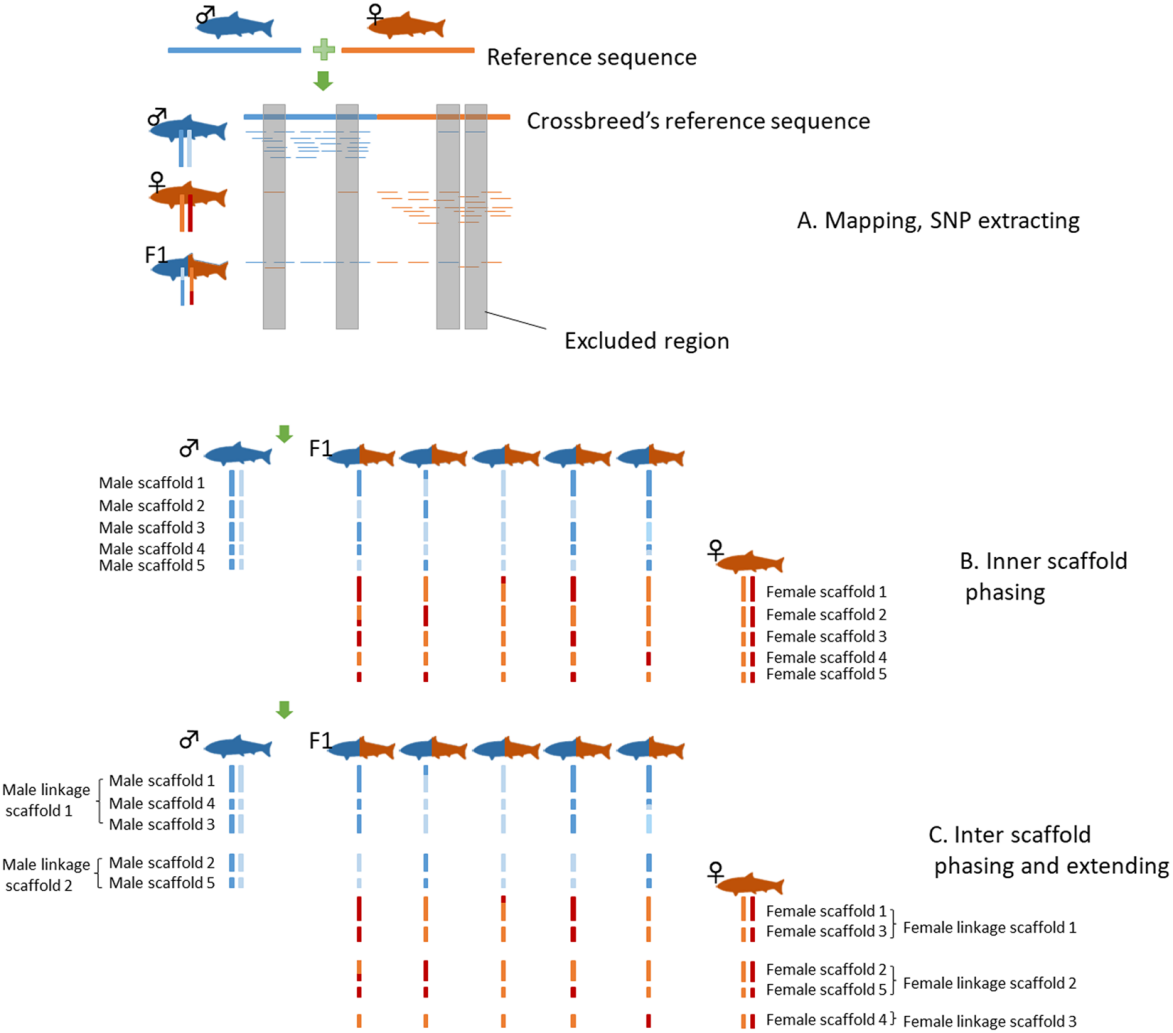
Overview of the SELDLA analysis flow. (**A**) Sequence data are mapped to a reference sequence obtained by combining parental genomes, and SNPs in specific regions are extracted. (**B**) To analyze the genome of a parent, we focus only on the reference sequence of that parent, determine which of its two chromosomes was passed to the hybrid, and phase. This is repeated from the beginning of the scaffold. (**C**) We arrange scaffolds with similar phase patterns on the chromosome. If phase patterns are different at the beginning and end of the scaffold, it is also possible to determine the orientation on the chromosome.

However, a single read of each polymorphic site is sufficient to determine the haplotype in haploid individuals, such as those generated by gynogenesis or androgenesis in some organisms (Fig. 1B), and each individual can thus be haplotyped with substantially fewer reads. The haplotype inherited from the diploid parent can then be inferred^9^. By extension, haplotyping is also very simple if each read can be clearly attributed to the mother or father (Fig. 2A). For example, when we find a read inherited from the mother, we need only determine either of the two maternal haplotypes, for which the required coverage is only one (Fig. 1C). By this strategy, independent linkage maps can be generated for both the mother and father (Figs 1C and 2C). However, maternal and paternal genomes are typically almost the same and are usually indistinguishable in the offspring.

To test this approach, we generated hybrid offspring from two interfertile species (Fig. 1C): torafugu, or tiger pufferfish (*Takifugu rubripes*), and gomafugu (*Takifugu stictonotus*). *T. rubripes* is suitable as a reference, as a draft genome and linkage map are already available. In contrast, only the mitochondrial genome is known for *T. stictonotus*. As the analysis is based on whole genomes, almost all SNPs are suitable as polymorphic markers, the density of which are typically higher in comparison to all others. Accordingly, the resolution of the resulting linkage map would be limited in theory not by the number of markers, but by the number of crossover sites.

The linkage maps thus obtained simultaneously for both species were high-resolution and high-coverage, especially in comparison to the latest draft sequence for *T. rubripes* (FUGU5). We also constructed the first linkage map and draft genome sequence for *T. stictonotus*, consisting of 22 chromosomes. We believe this is the first instance in which a draft sequence of the correct number of chromosomes was obtained in a single analysis. Our strategy should be applicable to most sexually reproducing organisms, and thus should facilitate genome analysis of not only oviparous organisms, but also viviparous animals and plants.

## Material and Methods

### Fish and artificial insemination

Eggs from a female *T. stictonotus* caught on the day of the experiment were artificially inseminated with stored sperm obtained from a *T. rubripes* male in 2014. Hybrid fry were collected individually 10 days after fertilization (4–6 days after hatching). Because we lost the actual source of eggs, we caught another *T. stictonotus* female for genome sequencing. All experiments were carried out in accordance with the IACUC (Institutional Animal Care and Use Committee) of the Graduate School of Agricultural and Life Sciences, University of Tokyo. All live fish were sacrificed by submersion in ice water for at least 10 minutes followed by decapitation. This study was approved by the subcommittee on institutional animal care and use of Graduate School of Agricultural and Life Sciences, The University of Tokyo (permission # P14-952).

### Library preparation and sequencing

Genomic DNA was individually extracted from 192 fry using DNeasy Blood and Tissue Kit (QIAGEN, Hilden, Germany). Libraries were prepared using Nextera DNA Sample Prep Kit (Illumina, San Diego, CA, USA) and Nextera Index Kit (Illumina). Libraries from 96 hybrid fry were then mixed at equal concentrations to obtain two pools (192 samples in total), which were processed and sequenced on a HiSeq. 2500 Sequencer (Illumina) to obtain paired-end sequences of 100 bp each. Four samples with few reads were excluded.

### Genome assembly

*T. rubripes* and *T. stictonotus* adults were also sequenced to generate reference sequences with which to identify the source of each raw read in hybrid fry, and to call and map SNPs. Raw data were obtained from one round of sequencing on HiSeq 2500. For *T. rubripes*, the stored sperm was used as source of paternal genomic DNA. For *T. stictonotus*, a second individual as described above was used as the source of a mother-equivalent genome. Reads were assembled in CLC Assembly Cell 5.0.3 (QIAGEN), with an insert size of 100–800 bp and default values for all other parameters.

### Seldla

We developed SELDLA, a novel data processing pipeline to construct a linkage map from genomic hybrids. SELDLA is available at http://www.suikou.fs.a.u-tokyo.ac.jp/software/SELDLA/, and is illustrated in Fig. 2. Briefly, we first combined the *T. rubripes* genome (FUGU4) and the *de novo T. stictonotus* genome assembled in this study. Reads from both adult fish and from 192 hybrid fry were then mapped onto the combined genome in BWA mem ver. 0.7.15, using “- t 4 - M” as the input parameter^10^. Uniquely mapped reads were extracted, sorted in samtools ver. 1.4^11^, and mined for SNPs in UnifiedGenotyper in GATK ver. 3.2-2^12^. SNPs were called with “- stand_emit_conf 0 - glm SNP,” even from just one read.

We excluded regions in the combined genome to which reads from both *T. stictonotus* and *T. rubripes* were mapped. We also excluded regions to which reads were mapped with more than four times the average depth. Each contig/scaffold was then phased, and linkage was measured by concordance rate of phases from all samples. In particular, the phase at two contigs/scaffolds was considered concordant when such contigs/scaffolds were completely linked, i.e., without crossover points, in 188 hybrids. In contrast, the concordance rate was close to 50% for two contigs/scaffolds that were not linked. We note that satisfactory scaffold elongation was achieved by setting the concordance rate threshold to 90% for male fish and to 70% for female fish, as recombination rates are lower in male pufferfish than in female pufferfish^5^.

### Comparative genome analysis

To generate Circos and dot plots, LAST ver. 861^13^ was used for homology searches. LAST results were converted to tab-delimited text using suitable scripts, and regions with continuous alignment of at least 1,000 bp were obtained. Circos plots were drawn in Circos ver. 0.69-5^14^ to compare the selected fragments against the 22 chromosomes in *T. rubripes* (FUGU5). All scaffolds were also compared in awk scripts, and dot plots were drawn in R based on LAST results.

### Linkage mapping

Physical distance was calculated directly by adding scaffold lengths. Linkage distance was calculated as follows. Each scaffold was located, ordered, and oriented using the phases of the 188 individuals at both ends of the scaffold. The linkage map was then constructed by sum up of the two-point linkage analysis between adjacent phases. To exclude false recombination due to noise, we eliminated phases that did not match continuously for three or more scaffolds in each individual.

## Results

### Sequencing of samples

We sequenced 192 hybrid fry, and obtained suitable data from 188. Sequence data were also obtained from the father, as well as from a mother-equivalent following loss of the mother. Sequence statistics are listed in Table 1. Assuming a genome size of 391 Mb for both species, coverage was 60.7- and 83.5-fold for *T. rubripes* and *T. stictonotus*, respectively. Because the size of the genome is doubled (782 Mb) in hybrids, coverage for each was 1.8-fold on average.

**Table 1:** Sequencing statistics for the male parent (*T. rubripes*), a female *T. stictonotus*, and hybrid fry.

		Reads	Bases (Gbp)	Fold
*Takifugu rubripes* (male)		118,907,833	23.8	60.7
*Takifugu stictonotus* (female)		163,351,727	32.7	83.5
Crossbreed (n = 188)	Average	7,003,042	1.4	1.8
	Min	2,678,368	0.5	0.7
	Max	134,959,206	27	34.5
Total		1,598,831,379	319.8	

To calculate depth, a genome size of 391 Mb was used for *T. rubripes* and *T. stictonotus*, as previously reported. The genome size was doubled to 782 Mb for hybrid fry.

### Reference genome assemblies

The N50 for the *de novo T. rubripes* genome that we obtained (de-novo-contigs-Tr) was 13.0 kb (Table 2), which is 66 times shorter than that of the previous version of the *T. rubripes* genome (FUGU4), for which the N50 was 858 kb. Therefore, FUGU4 was used as a reference in further analysis. For *T. stictonotus*, the genome that we obtained (de-novo-contigs-Ts), with N50 of 15.9 kb, was used as a reference because no other genome sequence is available.

**Table 2:** Assembly and scaffolding statistics for *de novo T. rubripes* and *T. stictonotus* genomes, as well as for FUGU4 and FUGU5, which are published *T. rubripes* reference genomes.

	N50 (bp)	Sum of scaffold/contig sizes (bp)	Max size of scaffolds/contigs (bp)	Number of scaffolds/contigs
*Takifugu rubripes* (*T. rubripes* scaffolds/contigs)	13,002	326,596,034	158,829	72,571
*Takifugu stictonotus* (*T. stictonotus* scaffolds/contigs)	15,925	336,757,840	164,291	78,881
FUGU4	858,115	393,296,343	7,245,445	7,213
FUGU5	11,516,971	391,484,715 (Located: 86%, Oriented: 72%)	23,260,604	6,835

N50, the shortest sequence length at 50% of the genome; sum, total scaffold length; max, maximum scaffold length; n, number of scaffolds. (*1) These data do not reflect the scaffolds/contigs located but not oriented.

### Extraction of SNP markers from very low-coverage data

We developed Scaffold Extender with Low Depth Linkage Analysis (SELDLA), an analysis pipeline (Fig. 2) with which we attempted to extend FUGU4 scaffolds using new low-coverage sequence data from hybrid fry. We then assembled the extended FUGU4 contigs/scaffolds according to the new SELDLA-derived linkage map, and compared the assembly with FUGU5, the current version of the *T. rubripes* genome, which was constructed by ordering FUGU4 contigs/scaffolds according to a linkage map of 1,222 microsatellites. We regarded the FUGU4 plus de-novo-contigs-Ts as a haploid reference sequence for a hybrid individual. FUGU4 and the de-novo-contigs-Ts genome were 98.1% homologous over aligned sequences throughout the genome. We then mapped each read of the hybrid individual to the FUGU4 plus de-novo-contigs-Ts genome using BWA mem^10^, and extracted those in which a continuous stretch of 90 bp or more (out of 100 bp) was mapped exclusively and uniquely. The average mapping rate after this process was 82.8%. Subsequently, SNPs were called in GATK UnifiedGenotyper^12^. We note that SNPs were called even if present in only one read, which is generally discarded as a sequence error in conventional analysis.

### Extraction of SNP markers from *T. rubripes*

We have previously reported a method of genotyping from low-coverage sequence data^9^. In the study, we sequenced double haploid individuals with a low depth of coverage (~1× ) and performed SNP typing. We divided the reference genome into 8 kb segments and determined the phases of the individual 8 kb segments based on the sets of SNP typing data in the segments to obtain the short segment genotype. We then obtained highly accurate typing results by imputation and correction based on the typing data of multiple SNPs contained in the individual 8 kb segments. In the present study, we further refined the previous method to determine highly accurate genotype information at both ends of the scaffold using whole SNPs in the scaffold (Supplementary Fig. 1 and Supplementary Information 1).

Linkage analysis was performed separately for *T. rubripes* and *T. stictonotus*. In the former, 3,846,895 SNPs were initially extracted. However, a site was regarded as highly similar between the two species and thus excluded from linkage analysis, if even a single *T. stictonotus* read was mapped to *T. rubripes* (Fig. 2A). As a result, only 1,293,394 SNPs remained for further processing. We plotted the ratios of indistinguishable SNPs between *T. rubripes* and *T. stictonotus* along the chromosomes of *T. rubripes* (Supplementary Fig. 2). Although the ratios of indistinguishable SNPs are diverse among sub-chromosomal regions, in any one sub-chromosomal region, a sufficient number of distinguishable SNPs were found. Theoretically, we should also be able to detect if such indistinguishable regions exist when observing larger linkage distance between two neighboring markers; however, we found no such region. To minimize potential errors, reads mapped with a depth of more than 4 times the average depth were then removed. Furthermore, we extracted informative SNPs confirmed to be heterozygous in the paternal genome, as every SNP found in paternal sequences in hybrid fry should also be found in the diploid genome of the father. After these steps, 606,110 markers were available for analysis. SNPs found in less than 10% of samples were excluded, along with those for which minor alleles were found in less than 30% of samples (major alleles found in more than 70% of samples). SNPs heterozygous in more than 20% of individuals were suspected to be multi-copy genes and were filtered out. Finally, SNPs that appeared to be a crossover breakpoint in a scaffold/contig of more than 10% of individuals were regarded as noise and were excluded. Ultimately, 442,723 markers were used in linkage analysis, for an average density of one marker per 903 bp.

### Elongation of FUGU4 scaffolds by SELDLA

Because algorithms to order and orient scaffolds are known to be NP-hard^15,16^, a normal heuristic approach was applied. A flowchart of the heuristic algorithm is shown in Supplementary Figs 1, 3 and 4. We have also added information about the principle of phasing in Supplementary Information 2. Briefly, each hybrid fry was phased from the beginning of a scaffold, and phase changes in the middle of the scaffold were listed as crossover points. After end-to-end phase patterns were collected from 188 individuals, scaffolds were sorted in descending order of length. Scaffolds with the most similar phase patterns were then connected to new linkage scaffolds. This process was repeated for the resulting connected scaffolds, until no test scaffold was found for which the similarity to a target scaffold was above the threshold value. If the phases at both ends of the newly connected scaffold were the same, the orientation of the scaffold could not be determined, and was filled with Ns (any of A, C, G, T bases) along the length of the unoriented scaffold.

Using 442,723 SNP markers, we phased all 188 *T. rubripes* genomes, as well as 4,513 of 7,213 scaffolds in FUGU4, such that phase was obtained for 95.7% of all nucleotides in the genome. Orientation was determined for 834 scaffolds representing 74.8% of nucleotides in the genome.

### Comparison of FUGU5 and SELDLA-extended FUGU4

**Figure 3:**
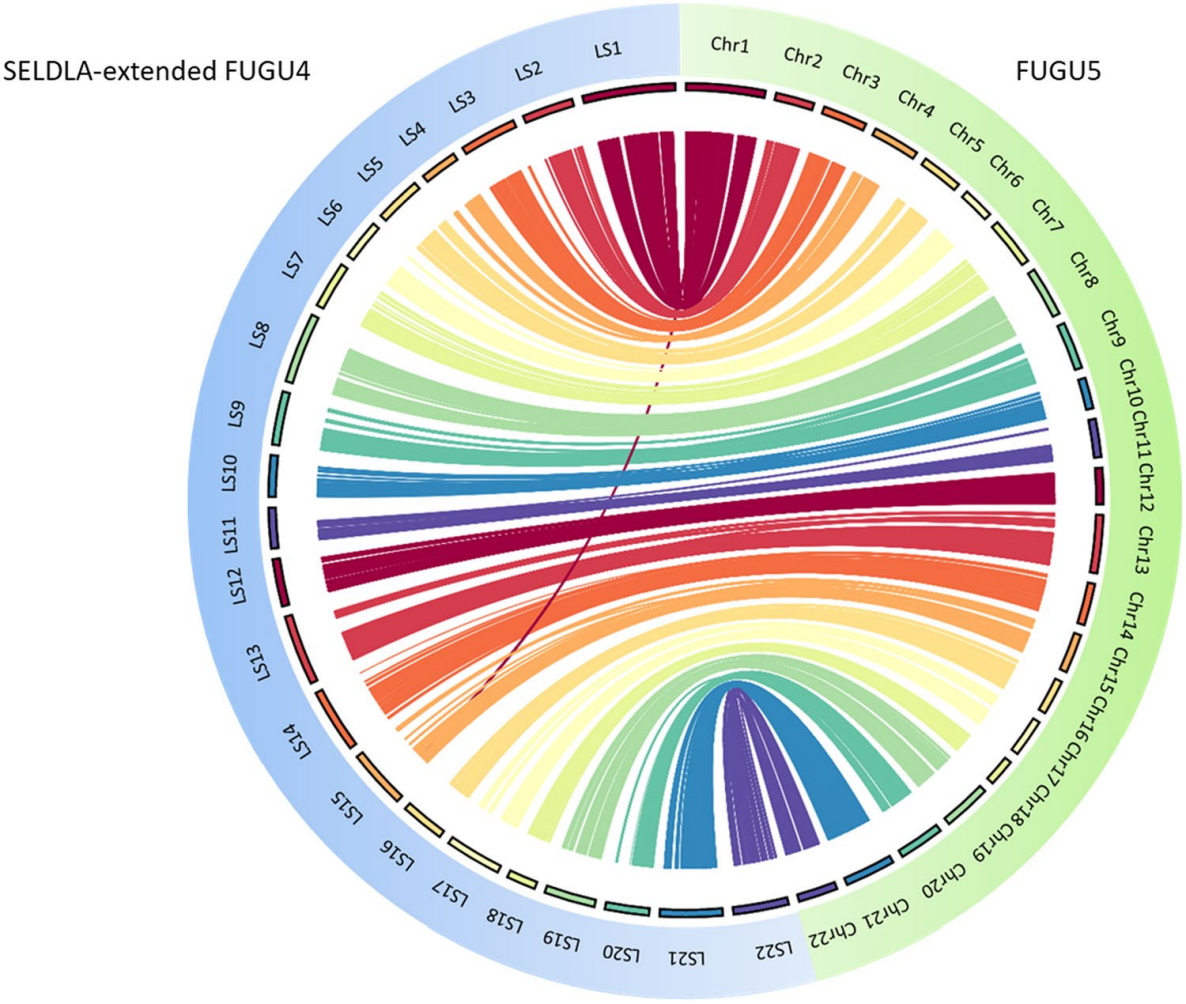
Circos comparison of 22 *T. rubripes* chromosomes between SELDLA-extended FUGU4 and FUGU5. Regions that could be aligned over 1 kb or more are connected by lines. LS: Linkage-group-extended (SELDLA-extended) Scaffold, chr: chromosome of FUGU5.

**Figure 4:**
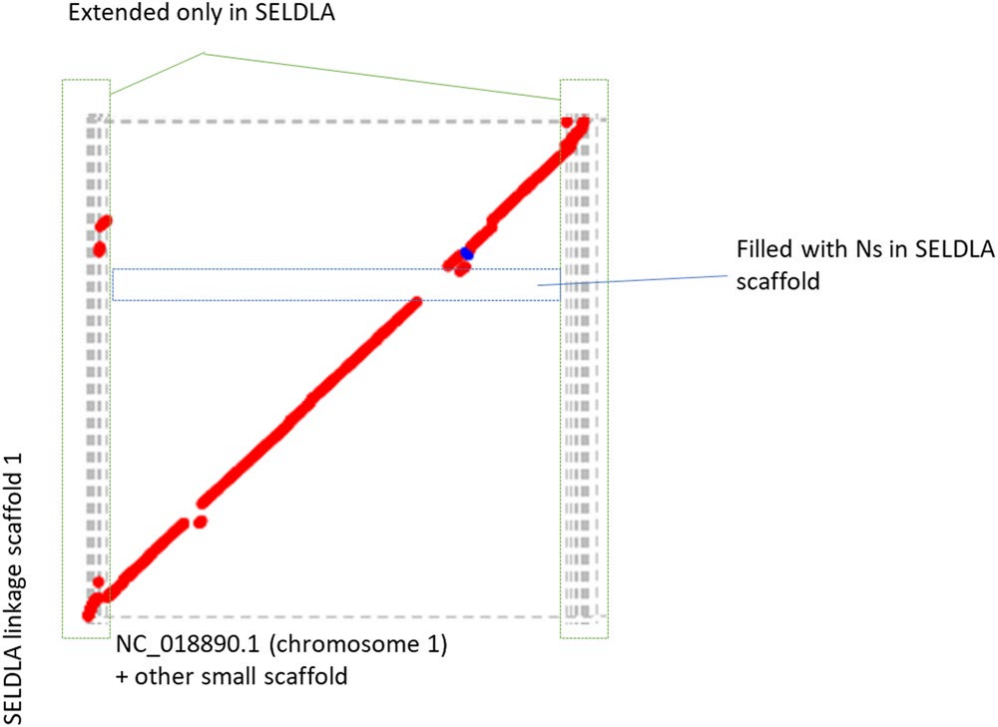
Dot plot homology comparison of chromosome 1 between SELDLA-extended FUGU4 and FUGU 5. Regions that could be aligned over 1 kb or more are plotted, with FUGU5 on the x-axis and SELDLA-extended FUGU4 on the y-axis. Red lines indicate alignment in the forward direction, whereas the blue line indicates alignment in the reverse direction. The plot shows that it was possible to arrange small FUGU5 scaffolds near telomeres according to the SELDLA-extended chromosome.

As listed in Table 3, extension by SELDLA increased the N50 in FUGU4 by 17-fold, such that it was now longer than that of FUGU5, which was generated by extending FUGU4 using conventional linkage analysis. A Circos plot (Fig. 3) confirmed that the genomic organization in SELDLA-extended FUGU4 is consistent with that of FUGU5 over 22 chromosomes, except at one site. Dot plots comparing all scaffolds between FUGU5 and SELDLA-extended FUGU4 are shown in Fig. 4 and Supplementary Fig. 5, and indicate that alignment was mostly successful throughout the entire genome. In addition, we found that many small scaffolds/contigs unmapped in the former were aligned at both ends to chromosomes in the latter (Fig. 4 and Supplementary Fig. 5), as clearly shown in a zoomed-in view of chromosome 1, a representative example. Indeed, several small scaffolds/contigs were incorporated and ordered into chromosome 1 in SELDLA-extended FUGU4, but not in FUGU5. This result implies that microsatellite markers near telomeric regions were inadequate in FUGU5 and highlights the large difference in marker density between microsatellites in FUGU5 and non-selected SNPs in this study. Several genomic inversions and translocations were also observed, suggesting structural polymorphisms between *T. rubripes* individuals. We note that these may cause erroneous ordering in SELDLA-extended FUGU4, FUGU5, or both. Finally, comparison of physical and linkage distance (Supplementary Fig. 6) indicated that linkage distance tends to be greater around telomeres, confirming that our result was consistent with that of a previous study^5^.

**Table 3:** Assembly and scaffolding statistics for the SELDLA-extended FUGU4 and SELDLA-extended *T. stictonotus* genome.

	N50 (bp)	Sum of scaffold/contig sizes (bp)	Max size of scaffolds/contigs (bp)	Number of scaffolds/contigs
SELDLA-extended FUGU4	15,004,475	420,596,343 (Located: 95.7%, Oriented: 74.8%)	27,254,446	4,483 (Located: 2,821, Oriented: 838 of FUGU4 scaffolds/contigs)
SELDLA-extended *T. stictonotus* scaffolds/contigs	33,840	413,277,840 (Located: 22.9%, Oriented: 7.7%)	11,477,030	71,229 (Located: 7,685, Oriented: 981 of *T. stictonotus* scaffolds/contigs)

### Scaffold elongation of the *T. stictonotus* genome by SELDLA

In addition, we also attempted to extend *T. stictonotus* contigs by SELDLA. These reference contigs were obtained from a female individual of *T. stictonotus* in a single round of paired-end sequencing on Illumina HiSeq 2500, followed by assembly in the CLC Assembly Cell 5.0.3 (https://www.qiagenbioinformatics.com/products/clc-assembly-cell/). The N50 for this genome was 15 kb. Although it is generally very difficult to perform linkage analysis using very short and numerous scaffolds/contigs, such analysis was possible for *T. stictonotus* due to the very high density of SNP markers.

In the process of SNP typing of *T. rubripes*, each SNP was found and typed concurrently. Misleading/false SNPs, which were not found to be heterozygous in the paternal genome, were removed from further analysis, because every SNP in the hybrid fry should be present in the paternal genome. The finding and typing of SNPs in *T. stictonotus* was performed in the same manner as in *T. rubripes*. However, we could not remove misleading/false SNPs using the methods used for *T. rubripes*, because we had lost the *T. stictonotus* female prior to extracting the genomic DNA, so we could not obtain information regarding the zygosity of maternal genome directly. Therefore, we omitted the removal step of misleading/false SNPs from further analysis of *T. stictonotus*.

Information regarding SNPs in the parental genome improves the accuracy of SNP typing of the offspring. We evaluated the influence of the omission of the removal step of misleading/false SNPs using *T. rubripes*. When we removed the misleading/false SNPs from the *T. rubripes* genome using the paternal zygote information, as described, the number of final SNPs was 442,723, but when we omitted the removal of misleading/false SNPs in the *T. rubripes* genome, the number of final SNPs was 443,302; this was a subtle difference (0.13%) that had little effect on the linkage map. We therefore consider that the existence of parental genome information is desirable but not essential for the analysis.

The number of extracted markers from *T. stictonotus* was 144,596, only 33% of the amount obtained from *T. rubripes*, perhaps reflecting closer kinship in *T. stictonotus*. Of the 78,881 scaffolds in the draft genome, phases were determined for 10,309, encompassing 22.9% of all nucleotides. These phased scaffolds were clustered in 22 linkage groups, corresponding to the number of chromosomes/linkage groups in *T. rubripes*. Orientation was also determined for 1,097 scaffolds, covering 7.7% of all nucleotides. The remaining 9,212 scaffolds/contigs were mapped to linkage groups, but in unknown orientations, mainly because there were no crossover points in the genome corresponding to short scaffolds/contigs from any of 188 individuals. In some cases, this effect might be due to lack of real SNPs. Surprisingly, the maximum scaffold length in the SELDLA-extended genome was 15.9 Mbp, which is 68% of the maximum scaffold length in FUGU5.

**Figure 5:**
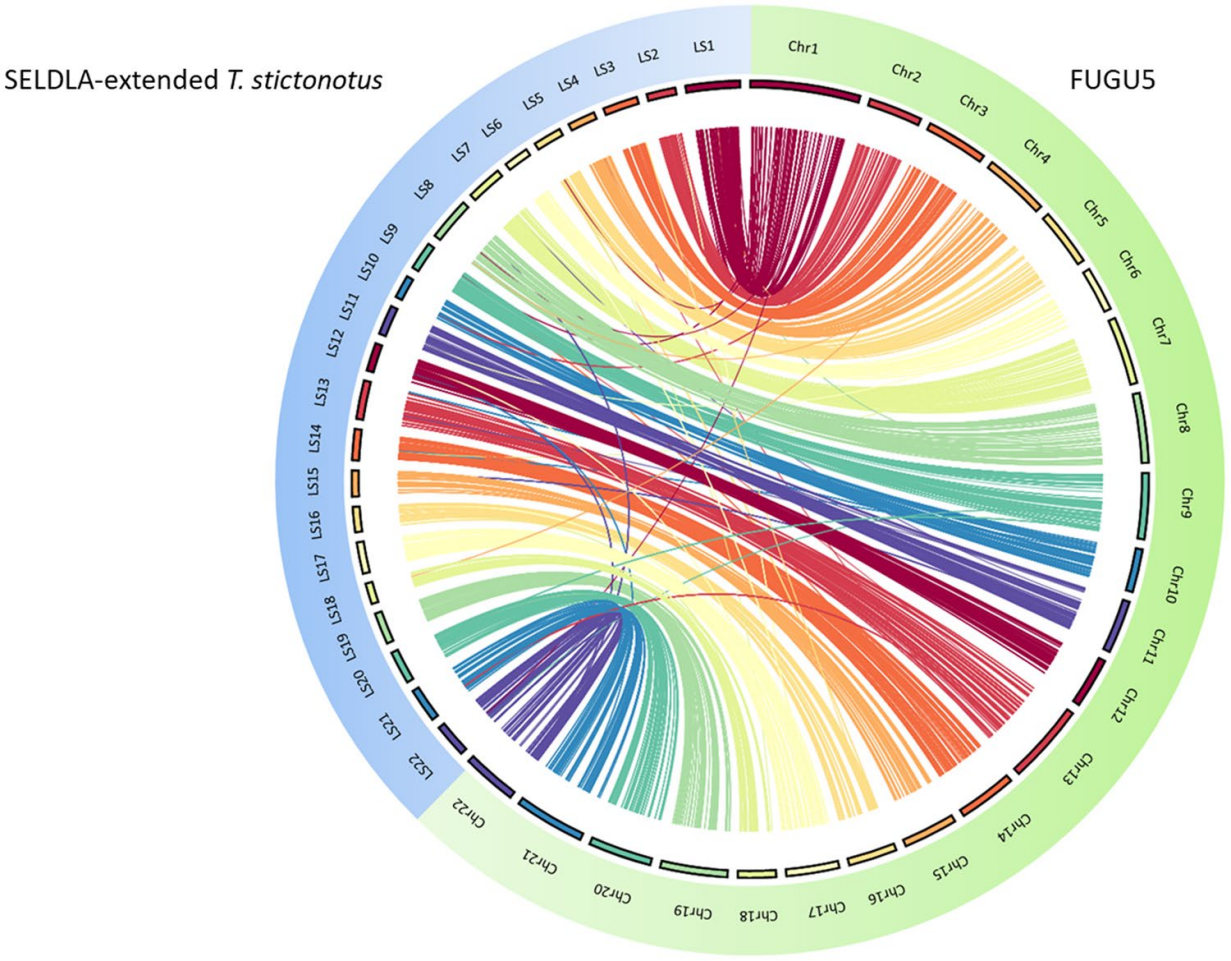
Comparison of FUGU5 and SELDLA-extended *T. stictonotus* genome. Regions that could be aligned over 1 kb or more in each of 22 chromosomes are connected by lines.

**Figure 6:**
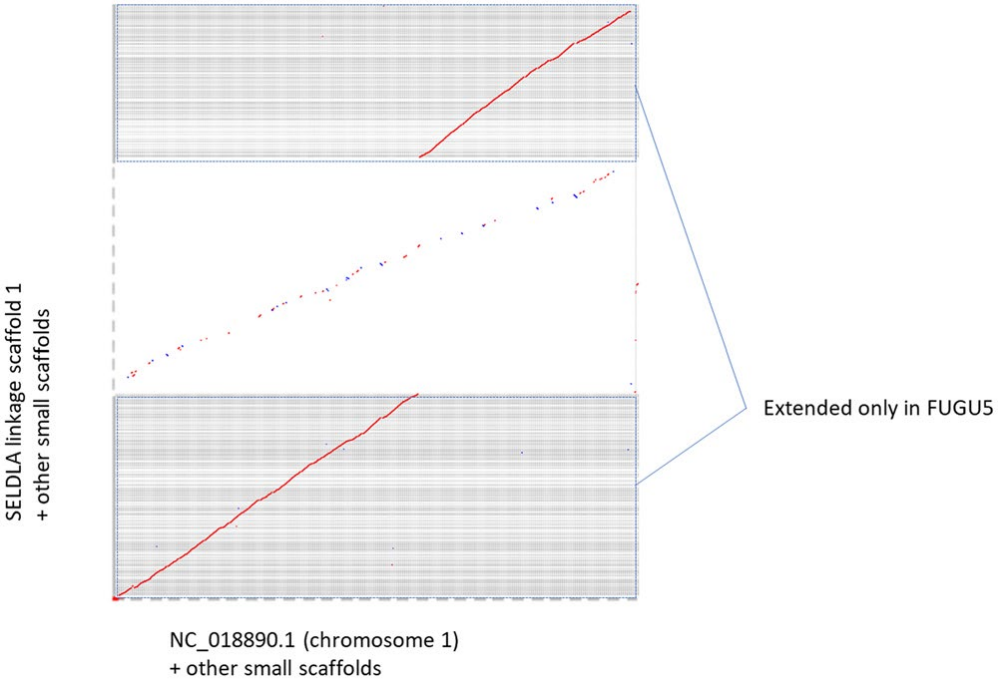
Dot plot homology comparison of chromosome 1 between FUGU 5 and SELDLA-extended *T. stictonotus* genome. Regions that could be aligned over 1 kb or more are plotted, with FUGU5 on the x-axis and SELDLA-extended *T. stictonotus* genome on the y-axis. Red lines indicate alignment in the forward direction, whereas blue lines indicate alignment in the reverse direction. LS: Linkage-group-extended (SELDLA-extended) Scaffold, chr: chromosome of FUGU5.

As *T. rubripes* and *T. stictonotus* belong to the same genus, the genome organization of both was expected to be similar. Indeed, a Circos plot of SELDLA-extended *T. stictonotus* and FUGU5 showed not only synteny, but also highly conserved sequence organization (Fig. 5). In addition, whole-genome dot plots (Fig. 6 and Supplementary Fig. 7) showed contiguous alignment over the entire chromosomes, although only 22.9% of nucleotides were phased. Physical and genetic distances in each SELDLA-extended *T. stictonotus* and FUGU5 are plotted in Supplementary Fig. 8.

## Discussion

We initially speculated that whole-genome sequencing would be suitable to type SNPs in double haploid and/or haploid organisms because a single read would be sufficient. We note that SNPs are generally useful as polymorphic markers not only because they are easy to type, but also because they are very common. In addition, we can compensate for missing data at a site in one individual because the phase is successive except around crossover points, of which there were, on average, 1.3 per chromosome 1 in *T. rubripes* and 3.6 per chromosome 1 in *T. stictonotus*. In contrast, typing of microsatellite polymorphic markers by conventional methods in double haploid and/or haploid organisms is less advantageous because these organisms contain only half of the information compared with diploids. Moreover, we speculated that if we can trace the origin of each read in an organism to either of its parents, linkage analysis for the father and mother can be performed independently. This process is essentially equivalent to simultaneous construction of linkage maps for two lines of double haploid/haploid organisms.

For our approach to be effective, the maternal and paternal genomes used for crosses must be distinguishable from each other. The similarity of the aligned region of paternal *T. rubripes* and maternal *T. stictonotus* parents in this study was 98.1%. Therefore, it should ideally be possible to generate linkage maps using the same approach as this study when the similarity of the two parents is the same or less than in this study. Our preliminarily study of pearl oyster indicated that our approach might be applicable even when using parents of the same species. The application of this approach depends on not only the similarity between the parental genomes, but also the lengths and accuracies of reads of sequencing. Therefore, advances in sequencing technology should widen the range of parental combinations. In support of this strategy, we also developed a new analytical pipeline, SELDLA, to generate high-resolution linkage maps, and, in parallel, to extend scaffolds/contigs based on thin, incomplete SNP data obtained from low-coverage genome data.

Following this approach, we mated two interfertile species, *T. rubripes* and *T. stictonotus*, to generate hybrid individuals with genes easily traceable to the parents. We succeeded in generating a new linkage map for *T. rubripes* from 442,723 SNPs in 188 hybrid individuals. The number of markers is significantly greater than the number of crossover points in the sperm of the father, which was estimated to be several thousand. Therefore, the resolution of the linkage map was limited not by the number of markers, but by the number of crossover sites. Indeed, the number of SNP markers used in this analysis is the highest among any other combination of markers and typing methods. We also succeeded in generating a draft genome of higher quality than the current draft (FUGU5). We demonstrate two comparisons of our approach with existing methods. The *T. rubripes* draft genome, FUGU5, was constructed by linkage analysis using 1,220 classical microsatellite markers, in which 86% of scaffolds in length was mapped to the genome, and 72% was oriented. Our method enabled us to map 95.7% of scaffolds in length and to orient 74.8% of the scaffolds, including those not previously located. In addition, we generated a linkage map for *T. stictonotus* for the first time, even with a very short N50 of 15 kb, mapping 22.9% of sequences to the genome.

To compare the costs of each method to determine the location, order, and orient scaffolds along chromosomes, we compared the number of reads required for genotyping per individual. In our study, we used 7.00 M reads per individual (Table 1) and utilized 587,319 (442,723 and 144,596) SNP markers for approximately 800 (400 + 400) Mb of genome sequence. The use of RAD-seq to construct linkage maps has previously required 7.28 M reads per individual (10,023 markers/1.8 Gb) in oil palm^17^, 6.70 M reads (9,968 SNP markers/423 Mb) in foxtail millet^18^, and 311.97 M reads (47,472 markers/1.1 Gb) in soybean^19^. This comparison suggests a benefit for the construction of a linkage map that has a higher precision and a lower comparative cost than RAD-seq.

We show in Fig. 4 that the genome we obtained can be extended, particularly at chromosome ends, which are problematic in FUGU5. Previously, the genetic length of chromosome 1 was reported as 99.7 cM and 179.9 cM for male and female *T. rubripes*, respectively^5^, but because we mapped many more scaffolds near telomeres, we have extended the genetic length of chromosome 1 to 124.3 cM for *T. rubripes* (male) (Supplementary Fig. 6) and 353.4 cM for *T. stictonotus* (female) (Supplementary Fig. 8), respectively. We suggest four possible explanations for the large difference in genetic distance between the *T. rubripes* male and *T*. *stictonotus* female. Our first suggestion is the inherent difference in genetic distance between males and females, which has been widely observed in humans (1.6 times^20^), zebrafish (2.74 times^21^), and sticklebacks (1.64 times^22^). Our previous study showed a genetic distance of about 2,300 cM in a *T. rubripes* female; however, the genetic distance of the *T. rubripes* female (2,300 cM) and the *T. stictonotus* female (3,900 cM) were both based on a single female individual. Therefore, the second possible reason for the difference between the *T. rubripes* male and *T. stictonotus* female is that the genetic distance may be variable among individuals. In humans, maternal age is a factor affecting crossover frequency^23^, but so far there is little data to discuss individual diversity of crossover frequency in other organisms. However, our method now enables us to construct the linkage maps of individuals easily, so we can address this very interesting point in the near future. The third possible cause of the difference in genetic distance of the *T. rubripes* male and *T. stictonotus* female is that they are different species. Finally, the fourth reason for the parental difference in genetic distance is the potential insufficient error correction and imputation of data due to shorter scaffolds with fewer SNPs before elongation in *T. stictonotus*. Of note, the reference sequence for SNP mapping was generated *de novo* by only one round of paired sequencing. However, combining a series of mate-pair and/or paired-end sequence data would generate a much longer N50, and enable precise and easy location of most longer scaffolds/contigs, as was achieved in *T. rubripes*.

PacBio and Oxford Nanopore sequencing have been recently demonstrated to generate much longer contiguous sequences^24,25^. Several new techniques to locate sequences in the genome, including 10× Genomics and Irys, have also been established^1,4^. These techniques also generate high-quality *de novo* genome sequences, and we anticipate that our strategy will prove to be very useful when combined with these new methods. More importantly, our method is based on genetic mapping, whereas others are based on physical mapping. We note that regardless of advances in physical methods, a genetic map would still be required to compare with and evaluate physical maps, as well as for various types of genetic analysis.

To save cost and labor, several genotyping methods based on a small number of polymorphic sites have been developed, including RAD-seq and genotyping-by-sequencing. If an inbred line is obtained by subrearing, the inbred individuals are essentially clones of the same haplotype, which can then be easily determined. However, this approach is limited to model organisms such as mice and zebrafish that can be inbred. Genotyping-by-sequencing with lower coverage has been employed for linkage map construction in several plants, and is a very effective strategy with inbred plant lines^26^, which are common in the field of plant genetics. However, if no inbred lines are available for the organism in question, it is time-consuming to establish new inbred lines for the construction of linkage maps. Similarly, generating double haploids/haploids is often difficult, and may also limit applicability. On the contrary, our method is based on mating interfertile species, and therefore should be more widely applicable. In our experience, torafugu embryos before hatching already provide sufficient DNA for genotyping, suggesting that hatching is not even necessary, which would further broaden the mating possibilities.

Thus, our method is one of the most effective and promising approaches for obtaining genomes from organisms that produce many eggs or seeds in one generation, such as fish, mollusks, amphibians, insects, and plants. In addition, we may even be able to apply our method to viviparous organisms via *in vitro* fertilization and/or differentiation of induced pluripotent cells into germ cells. Accordingly, we are now applying or planning to apply our method to obtain genomes from various organisms.

### Accession Numbers

WGS data has been deposited with the DNA Data Bank of Japan under accession number DRA006412.

## Electronic supplementary material

Supplementary Information

## Data Availability

The code for the SELDLA pipeline can be found at http://www.suikou.fs.a.u-tokyo.ac.jp/software/SELDLA/.
