# Supplementary material for: Artificially designed hybrids facilitate efficient generation of high-resolution linkage maps
Source: Sci Rep. 2018 Oct 31;8:16104. doi: 10.1038/s41598-018-34431-6 (PMC6208418; doi:10.1038/s41598-018-34431-6)
Supplement: Supplementary file 1 — Supplementary Information [file 41598_2018_34431_MOESM1_ESM.pdf]

Artificially designed hybrids facilitate efficient generation of high-resolution linkage maps

Kazutoshi Yoshitake[1], Yoji Igarashi[1], Misaki Mizukoshi[1], Shigeharu Kinoshita[1], Susumu Mitsuyama[1], Yutaka Suzuki[2], Kazuyoshi Saito[3], Shugo Watabe[4], Shuichi Asakawa[1,*]

Supplementary Information 1 The basic logic of correction or imputation of low-coverage data.

The basic logic is the correction of unlikely data, or imputation, which is based on the fact that the phases of parental homologous chromosomes change only at the sites of crossover. For example, here "|" indicates the site of crossover. If no crossover occurred between parental chromosomes, the phase is:

AAAAAAAAAAAAAAAAAAAAAAAAAAAAAAAAAAAAAAAAAAAAAAAAAAAAAAAAAAA
or
BBBBBBBBBBBBBBBBBBBBBBBBBBBBBBBBBBBBBBBBBBBBBBBBBBBBBBBBBBB.

If a crossover occurred, the phase is:
AAAAAAAAAAAAAAAAAAAAAAAAAAAAAAAAAA|BBBBBBBBBBBBBBBBBBBBBBBBB.

If some data are lacking, we can impute them.
From
AAAA-A---AAA-AAAAAA--AAAAAA-AA--AA|BBBB-B-BB-B-BBB--BBB-BBBB
to
AAAAAAAAAAAAAAAAAAAAAAAAAAAAAAAAAA|BBBBBBBBBBBBBBBBBBBBBBBBB.

Regarding correction:
Two or more crossovers are possible, as in the case below.
AAAAAAAAAAAAAAAAAA|BBBBBBBBBBBBBBBBBBBBBBB|AAAAAAAAAAAAAAAAAAA.

However, it is unlikely that two or more crossovers occurred within several bp - several kb, as in the case below.
AAAAAAAAAAAAAAAAAAAAAAAAAAAAAA|B|AAAAAAAAAAAAAAAAAAAAAAAAAAAAAA.
In such a case, we can correct them to
AAAAAAAAAAAAAAAAAAAAAAAAAAAAAAAAAAAAAAAAAAAAAAAAAAAAAAAAAAAAAA.

In addition, as a major premise of such correction, most errors occur at sites away from the SNP sites, randomly; therefore, we can omit them. For example, if a site shows a certain frequency rate (A:B = 46:54), we recognize it as an SNP site. If another site shows a rate of (A:B = 1:99), we can conclude that the site is not an SNP site, but a site caused by sequence error.

Supplementary Information 2 The principle of phasing of low-coverage data.

We show a description of the principle of phasing below:

Here, we assume in the below example.
0123456789: 10 SNP sites along a chromosome.
AAAAAAAAAA: Haplotype of one of the homologous chromosomes of the father.
BBBBBBBBBB: Haplotype of the other homologous chromosomes of the father.
CCCCCCCCCC: Haplotype of the reference genome sequence (not necessarily the father's one).

Assume an example of some SNP typing results, as below,
0:A≠C, B=C
1:A≠C, B≠C
2:A≠C, B=C
3:A=C, B≠C
4:A=C, B≠C
5:A≠C, B≠C
6:A≠C, B≠C
7:A≠C, B=C
8:A=C, B≠C
9:A≠C, B=C

We omit sets of typing results, " A≠C, B≠C", (but A=B, in biallelic sites), because such sites do not contribute to the linkage analysis. The remaining sites are shown below.
0:A≠C, B=C
2:A≠C, B=C
3:A=C, B≠C
4:A=C, B≠C
7:A≠C, B=C
8:A=C, B≠C
9:A≠C, B=C

If an offspring inherits the complete "A" chromosome (this means no crossover with "B"), the typing results are shown as below, where C indicates the same SNP type as the reference, and c indicates a different SNP type compared to the reference.
0:c

2:c
3:C
4:C
7:c
8:C
9:c

From now on, we describe them as ccCCcCc.

Similarly, if an offspring inherits the complete "B" chromosome, the typing results are shown as CCccCcC.

If offspring inherits chromosomes that are generated after one crossover, then the possible typing results of the offspring are shown as below.

cCccCcC
ccccCcC
ccCcCcC
ccCCCcC
ccCCccC
ccCCcCC
CcCCcCc
CCCCcCc
CCcCcCc
CCcccCc
CCccCCc
CCccCcc

We assume that the original haplotypes of two homologous chromosomes of the father are 0123456478: AAAAAAAAAA and BBBBBBBBBB, but at this point, we do not know those haplotypes.
Then, we try to identify the original haplotypes of A and B. More precisely, we try to identify the original haplotypes of 0234789, but not 0123456789, because we omit the sites of "A≠C, B≠C," as described.
When we focus on **sites 1 and 2**, four possible combinations are considered
Type 1; A:CC, B:cc

Type 2; A:Cc, B:cC

Type 3; A:cC, B:Cc

Type 4; A:cc, B:CC

Here, we can degenerate the types 1 & 4, and types 2 & 3, because we merely need to know which of the following is true: “one type is CC and the other type is cc” or “one type is Cc and the other type is cC.”

As is evident from the one-crossover example listed above, Cc and cC are rare (1/12, 1/12, respectively) and CC and cc (5/12, 5/12, respectively) are major (5:1). Therefore, we can identify that “CC and cc” at **sites 1 and 2** are the parental phases. When the marker density is high, as it is in our study, the ratio of major cases and rare cases is usually 100:1 or more (also depending on number of samples), so we can easily and confidently identify the original phases of the two neighboring sites of the parents.

Similarly, we can confidently identify the phases of 2 and 3, 3 and 4, and so on. Finally, we can identify the entire phase of each chromosome of the parents.

In this way, we identify that AAAAAAA is ccCCcCc, and BBBBBBB is CCccCcC.
(or AAAAAAA is CCccCcC, and BBBBBBB is ccCCcCc). This degeneration does not affect the linkage analysis; that is, we need not know which are from the grandfather and grandmother for the construction of the linkage map.

This is the explanation for the father, and we can demonstrate the same explanation in the case of the mother.

Supplementary Figure 1 Flowchart for determining the phases of both ends of a scaffold. (A) Input raw SNP data. Observed genotypes are “C” and “c”. (B) To minimize potential errors, remove reads mapped with a depth of more than 4 times the average depth. We extract SNPs confirmed to be heterozygous in the paternal genome, as every SNP found in paternal sequences in hybrid fry should also be found in the diploid genome of the father. After these steps, SNPs found in less than 10 % of samples were excluded, along with those for which minor alleles were found in less than 30 % of samples (major alleles are those found in more than 70 % of samples). SNPs heterozygous in more than 20 % of individuals were suspected to be multi-copy genes and were also filtered out. (C) Perform phasing and imputation in each scaffold. Phased genotypes are “A” and “B”. We searched SNPs from the beginning of scaffold and complemented the missing SNPs with previous SNPs. It was possible to avoid incorrect determination of crossover due to noise by approving crossover only if the same genotype appears three times or more. (D) Perform imputation in the opposite direction. (E) Determine the phased genotypes of each scaffold at both ends.

Supplementary Figure 2 The ratio of indistinguishable SNPs. The scaffolds of FUGU4 larger than 1 Mbp were separated by 1 Mbp and the ratio of SNPs not specific for *T. rubripes* was calculated in each region. Then, the calculated ratio was plotted on the position in the FUGU5 genome corresponding to the scaffold of FUGU4.

Supplementary Figure 3 Scaffold extension using the genotypes of both ends of each scaffold. (A) The list of all genotypes of both ends of each phased scaffold. (B) A matrix of the matching rates of genotypes at both ends of each scaffold. The end of scaffold with the highest matching rate is connected in order. (C) The result of inter-scaffold phasing and scaffold extension

Supplementary Figure 4 Relationship between genome, crossover points, and scaffolds. Black horizontal lines indicate an arbitrary area of the genome. Blue vertical lines indicate parental crossover points in any of the progeny. Red arrowed lines indicate scaffolds overlapping the crossover points, locating and orientating the scaffolds. Red lines within an ellipse indicate scaffolds that contain a sufficient number of informative SNPs and are located to the same genomic region between two crossover points, but are not overlapping the crossover points. Such scaffolds are not oriented and the relative order of them is not determined. The dotted red lines indicate scaffolds that contains no or an insufficient number of informative SNPs. Such scaffolds are not located.

Supplementary Figure 5 Dot plot homology comparison of all chromosomes between SELDLA-extended FUGU4 and FUGU 5. Regions that could be aligned over 1 kb or more are plotted, with FUGU5 on the x-axis and SELDLA-extended FUGU4 on the y-axis. Red lines indicate alignment in the forward direction, whereas blue lines indicate alignment in the reverse direction.

Supplementary Figure 6 Physical (Mb) and genetic (cM) distances in each SELDLA-extended *T. rubripes* chromosome. LS: Linkage-group-extended Scaffold corresponding to the same chromosome number of FUGU5. Lines indicating the corresponding between the physical map and the genetic map are shown at both ends of each scaffold.

Supplementary Figure 7 Dot plot homology comparison of all chromosomes between FUGU 5 and SELDLA-extended *T. stictonotus* genome. Regions that could be aligned over 1 kb or more are plotted, with FUGU5 on the x-axis and SELDLA-extended *T. stictonotus* genome on the y-axis. Red lines indicate alignment in the forward direction, whereas blue lines indicate alignment in the reverse direction.

Supplementary Figure 8 Physical (Mb) and genetic (cM) distances in each SELDLA-extended *T. stictonotus* chromosome. LS: Linkage-group-extended Scaffold corresponding to the same chromosome number of FUGU5. Lines indicating the correspondence between the physical map and the genetic map are shown at both ends of each scaffold. The average scaffold size of *T. stictonotus* is much shorter than that of *T. rubripes*; therefore, more lines are evident on the chromosome.

A) raw SNP data

ID	1	2	3	4	5	6	…
scaf1:1	C	.	c	.	C	.	
scaf1:2	c	C	.	.	c	.	
scaf1:3	C	C	C	C	C	.	
scaf1:4	C	.	.	.	.	.	
scaf1:5	c	.	C	C	.	c	
scaf1:6	.	c	c	.	C	C	
scaf1:7	c	c	.	c	.	c	
scaf1:8	c	C	c	.	.	c	
scaf1:9	.	.	.	c	.	.	
scaf1:10	.	C	c	.	c	c	
scaf1:11	C	c	C	.	.	C	

B) cleaned SNP data

ID	1	2	3	4	5	6	…
scaf1:1	C	.	c	.	C	.	
scaf1:2	c	C	.	.	c	.	
scaf1:5	c	.	C	C	.	c	
scaf1:6	.	c	c	.	C	C	
scaf1:8	c	C	c	.	.	c	
scaf1:10	.	C	c	.	c	c	
scaf1:11	C	c	C	.	.	C	

C) inner scaffold phasing and forward imputati

ID	1	2	3	4	5	6	…
scaf1:1	A	.	B	.	A	.	
scaf1:2	A	B	B	.	A	.	
scaf1:5	A	B	B	B	A	A	
scaf1:6	A	B	B	B	A	A	
scaf1:8	A	B	A	B	A	A	
scaf1:10	A	B	A	B	A	A	
scaf1:11	A	B	A	B	A	A	

D) backward imputation

ID	1	2	3	4	5	6	…
scaf1:1	A	B	B	B	A	A	
scaf1:2	A	B	B	B	A	A	
scaf1:5	A	B	B	B	A	A	
scaf1:6	A	B	B	B	A	A	
scaf1:8	A	B	A	B	A	A	
scaf1:10	A	B	A	B	A	A	
scaf1:11	A	B	A	B	A	A	

crossover point

E) genotypes of both ends of a phased scaffold

ID	1	2	3	4	5	6	…
scaf1:begin	A	B	B	B	A	B	
scaf1:end	A	B	A	B	A	A	

Supplementary Fig. 1

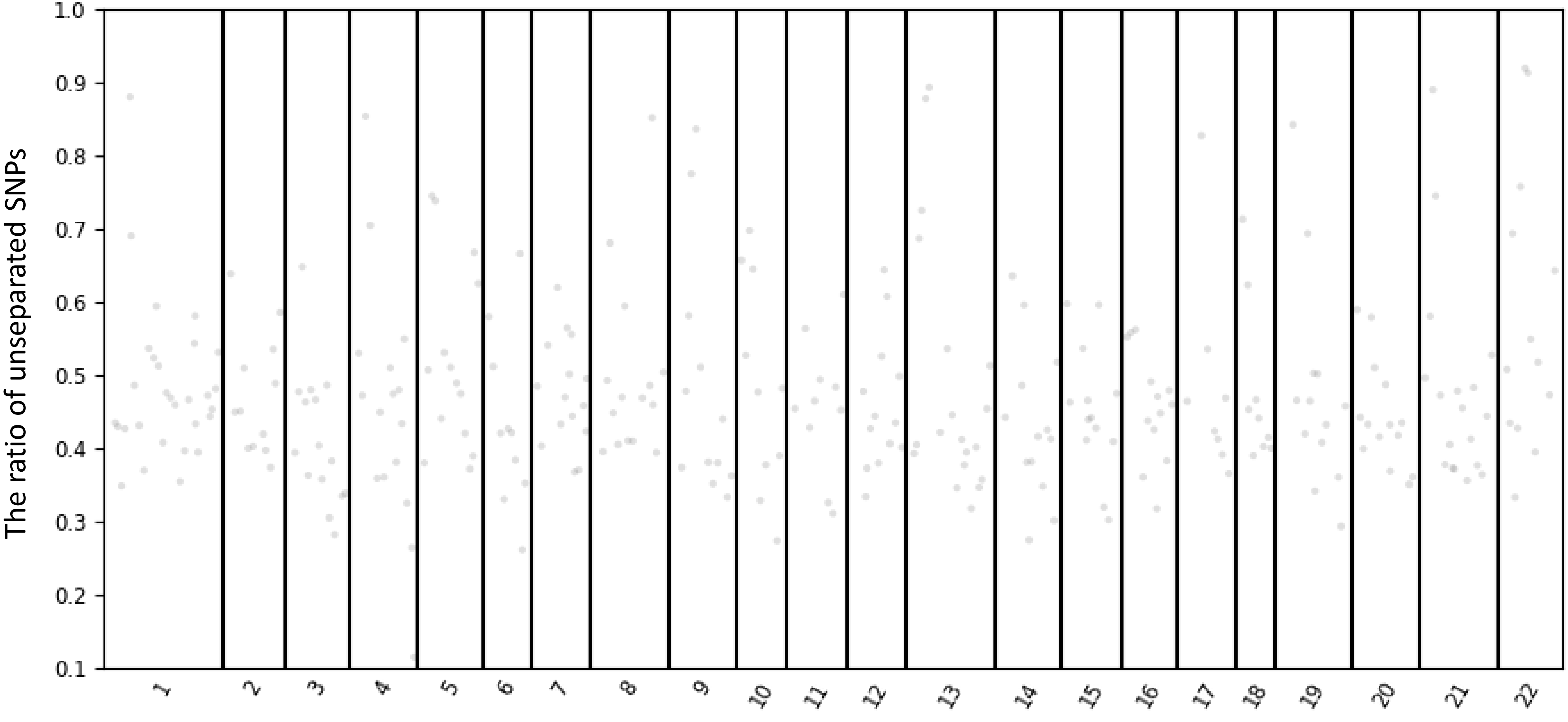

Supplementary Fig. 2

A) all genotypes of both ends of phased scaffolds

ID	1	2	3	4	5	6	…
scaf1:begin	C	c	c	c	C	c	
scaf1:end	C	c	C	c	C	C	
scaf2:begin	c	C	c	c	c	C	
scaf2:end	c	C	c	c	C	C	
scaf3:begin	c	C	c	C	c	c	
scaf3:end	c	C	C	C	c	c	
scaf4:begin	C	c	c	c	C	C	
scaf4:end	C	c	c	c	C	C	
scaf5:begin	c	C	c	c	c	C	
scaf5:end	c	C	C	C	c	C	

B) matching rate of genotypes at both ends

ID	scaf1: begin	scaf1: end	scaf2: begin	scaf2: end	scaf3: begin	scaf3: end	scaf4: begin	scaf4: end	scaf5: begin	scaf5: end
scaf1: begin		0.67	0.67	0.5	0.67	0.83	0.83	0.83	0.67	1
scaf1: end			0.67	0.5	1	0.83	0.83	0.83	0.67	0.67
scaf2: begin				0.83	0.67	0.5	0.5	0.5	1	0.67
scaf2: end					0.5	0.67	0.67	0.67	0.83	0.5
scaf3: begin						0.83	0.83	0.83	0.67	0.67
scaf3: end							1	1	0.5	0.83
scaf4: begin								1	0.5	0.83
scaf4: end									0.5	0.83
scaf5: begin										0.67
scaf5: end										

Supplementary Fig. 3

C) inter scaffold phasing and scaffold extension

ID	1	2	3	4	5	6	…
scaf2:end	A	B	A	A	B	B	located and oriented (-)
scaf2:begin	A	B	A	A	A	B	
scaf5:begin	A	B	A	A	A	B	located and oriented (+)
scaf5:end	A	B	B	B	A	B	
scaf1:begin	A	B	B	B	A	B	located and oriented (+)
scaf1:end	A	B	A	B	A	A	
scaf3:begin	A	B	A	B	A	A	located and oriented (+)
scaf3:end	A	B	B	B	A	A	
scaf4:begin	A	B	B	B	A	A	located but unoriented
scaf4:end	A	B	B	B	A	A	

Supplementary Fig. 3

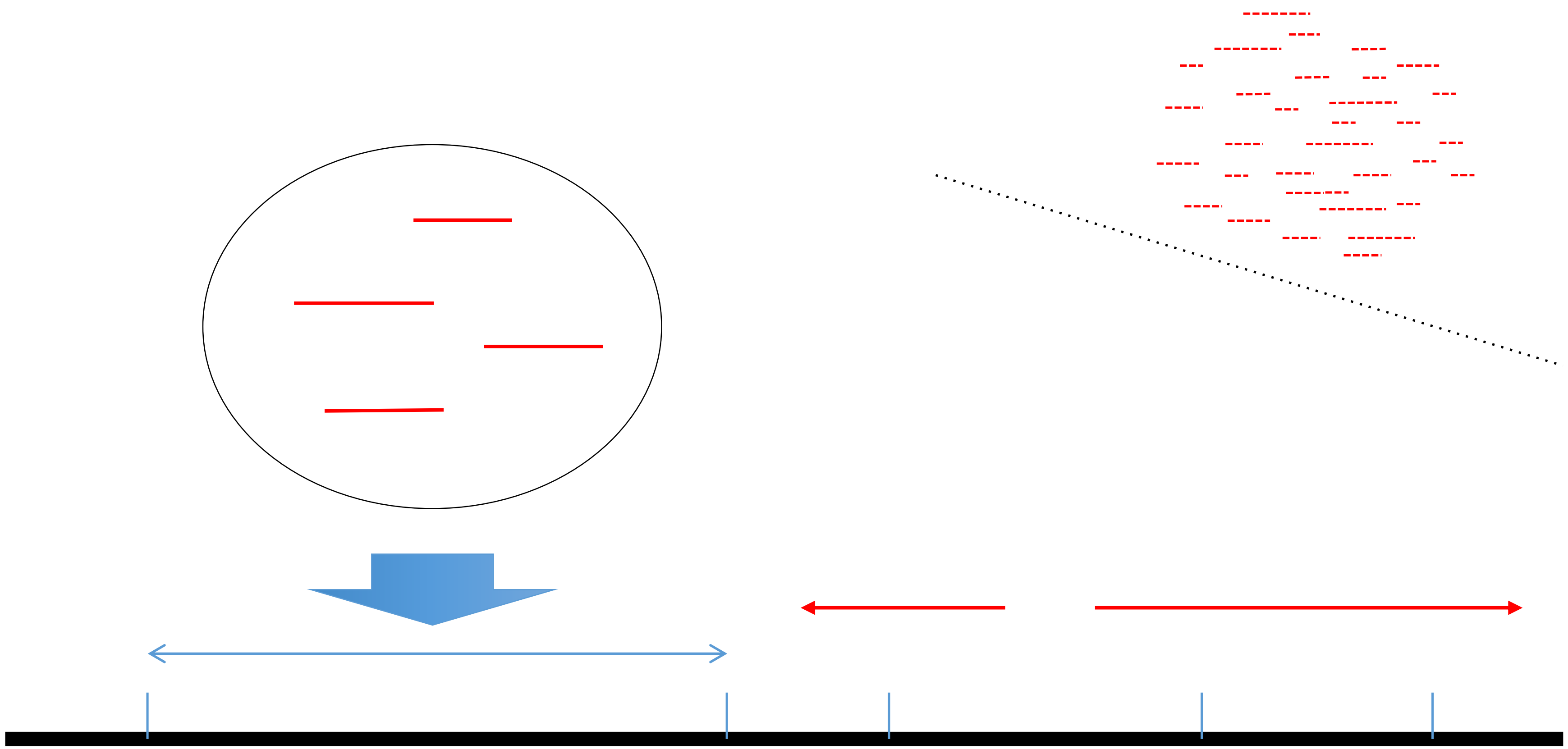

Supplementary Fig. 4

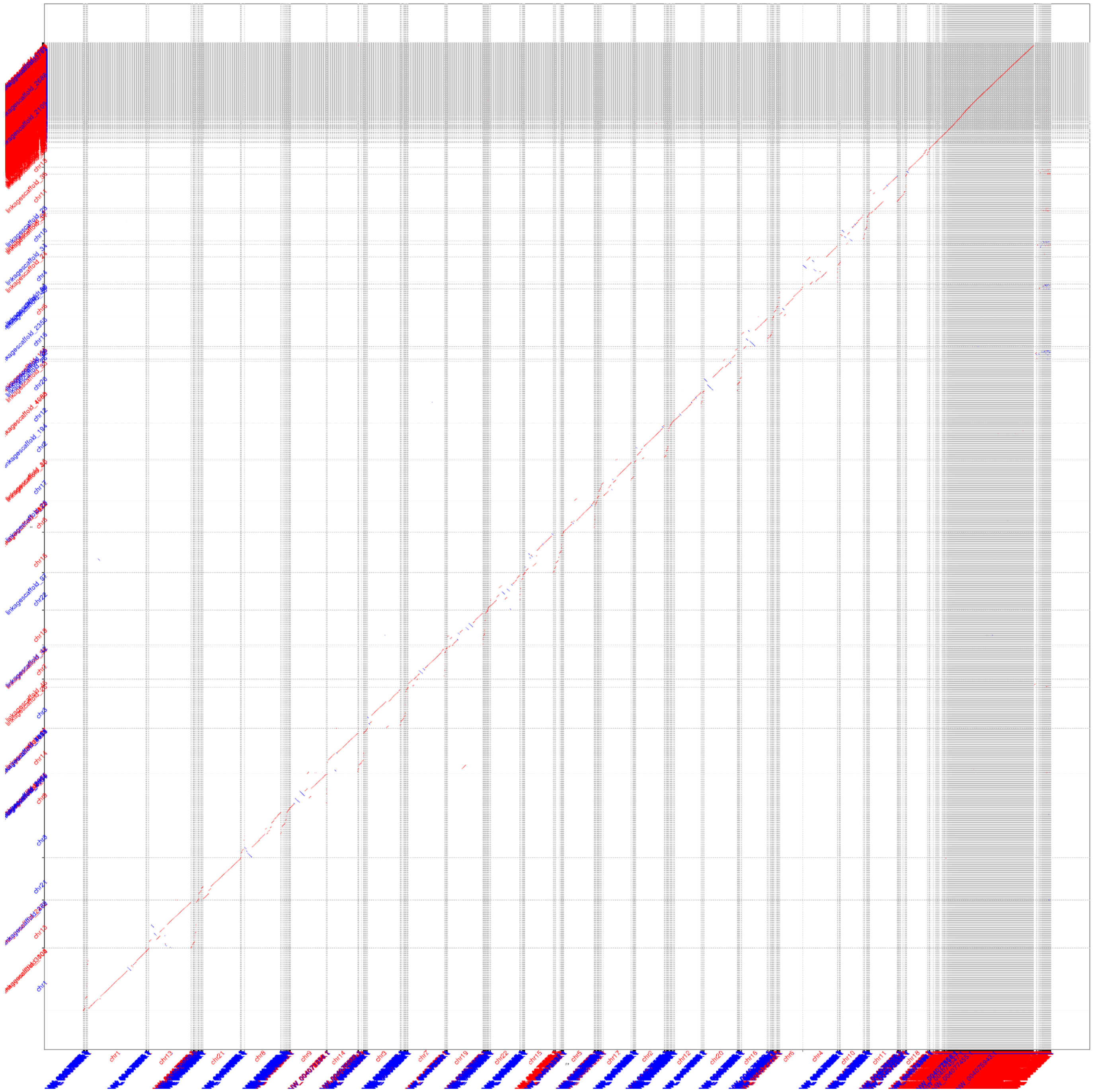

Supplementary Fig. 5 Comparison of total chromosomes of SELDLA-extended FUGU4 and FUGU5

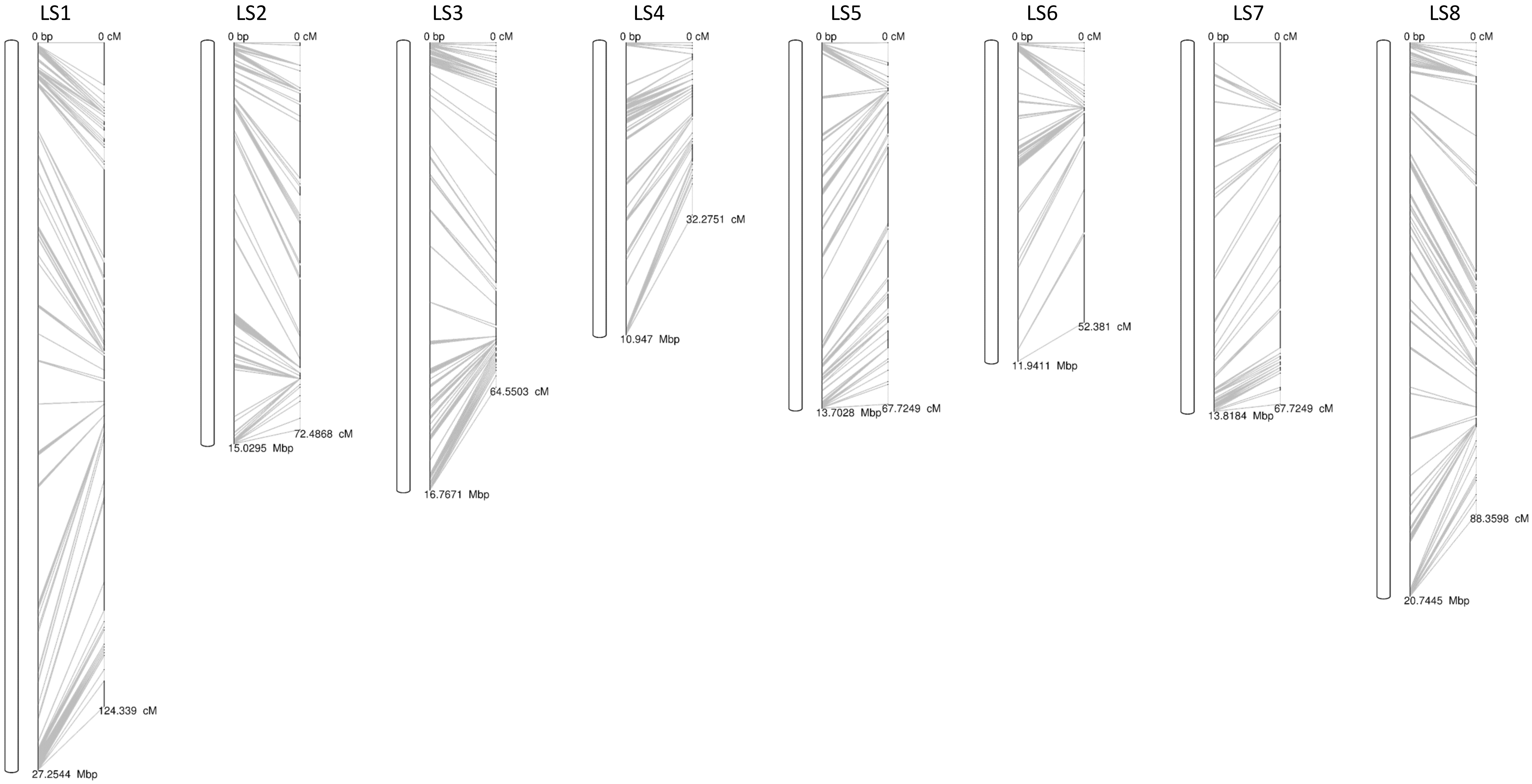

Supplementary Fig. 6 Linkage Map of SELDLA-extended FUGU4

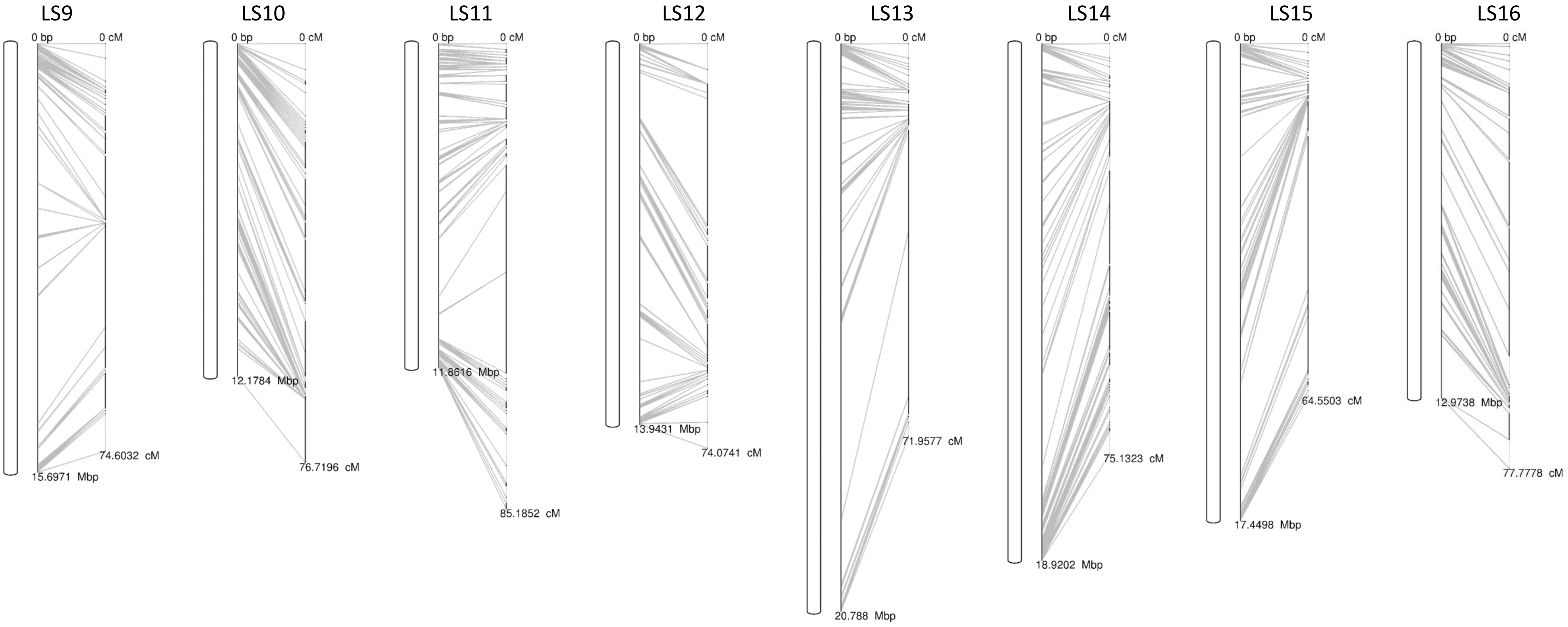

Supplementary Fig. 6 Linkage Map of SELDLA-extended FUGU4

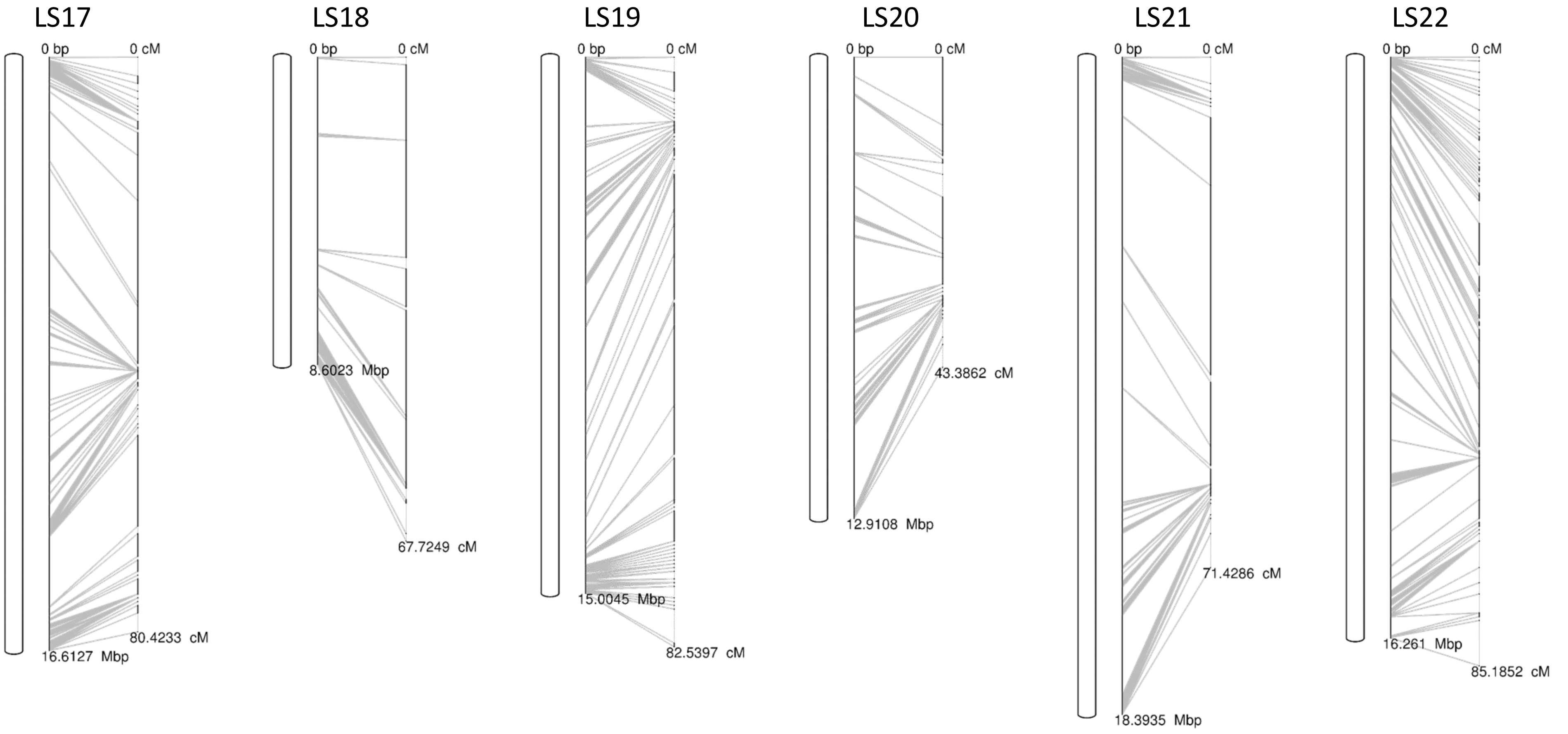

Supplementary Fig. 6 Linkage Map of SELDLA-extended FUGU4

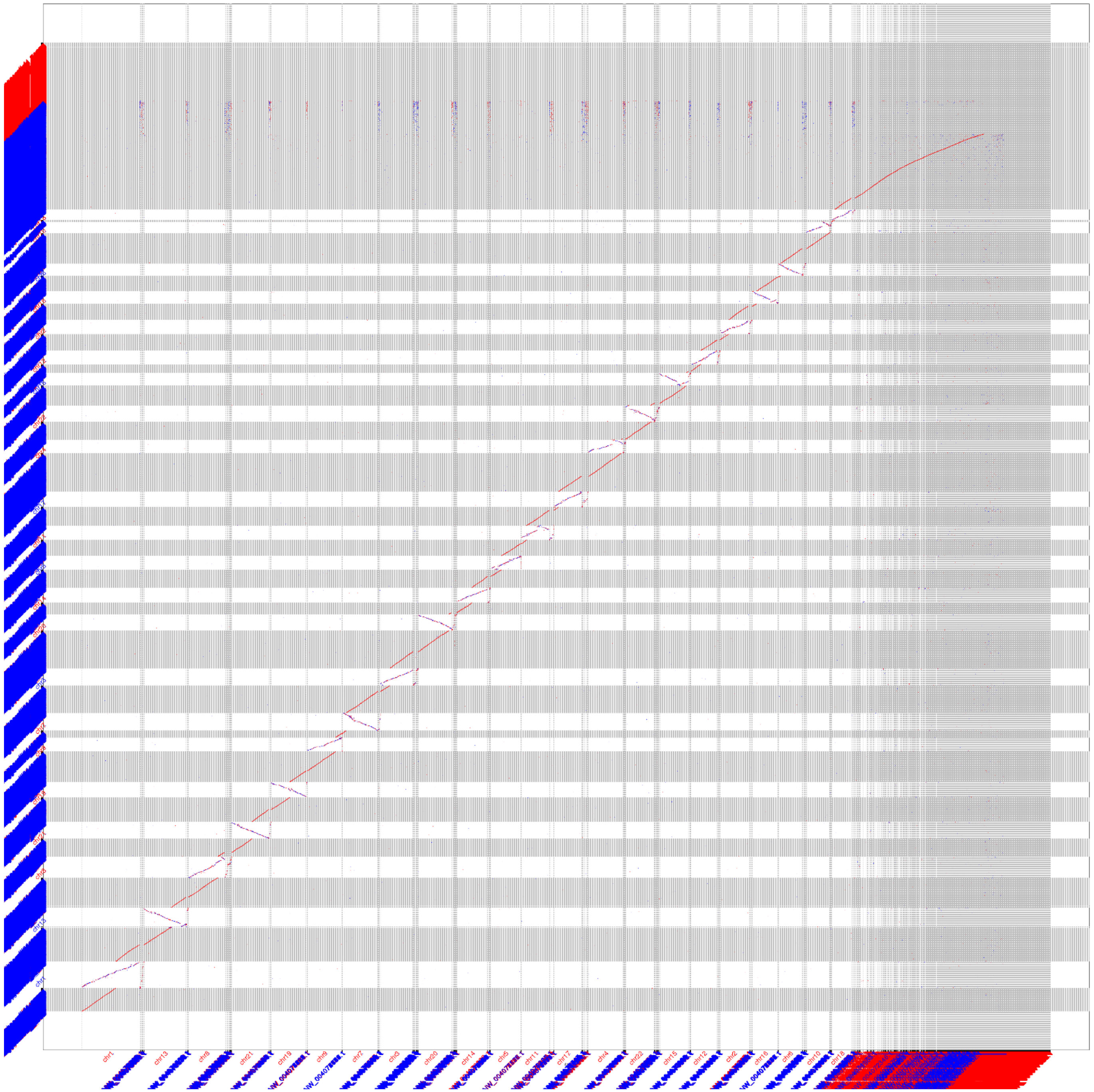

Supplementary Fig. 7 Comparison of total chromosomes of SELDLA-extended *T. stictonotus* and FUGU5

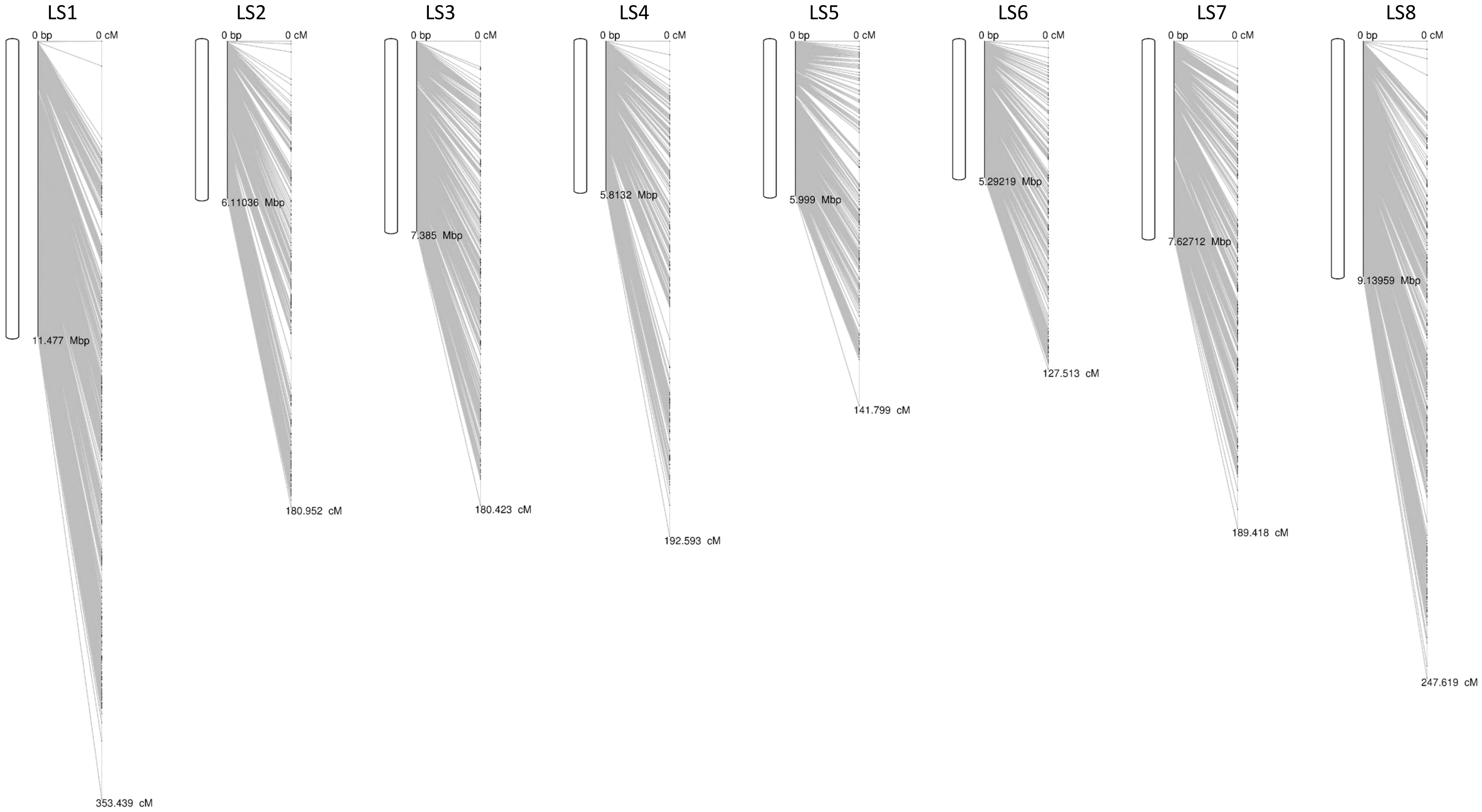

Supplementary Fig. 8 Linkage Map of SELDLA-extended *T. stictonotus*

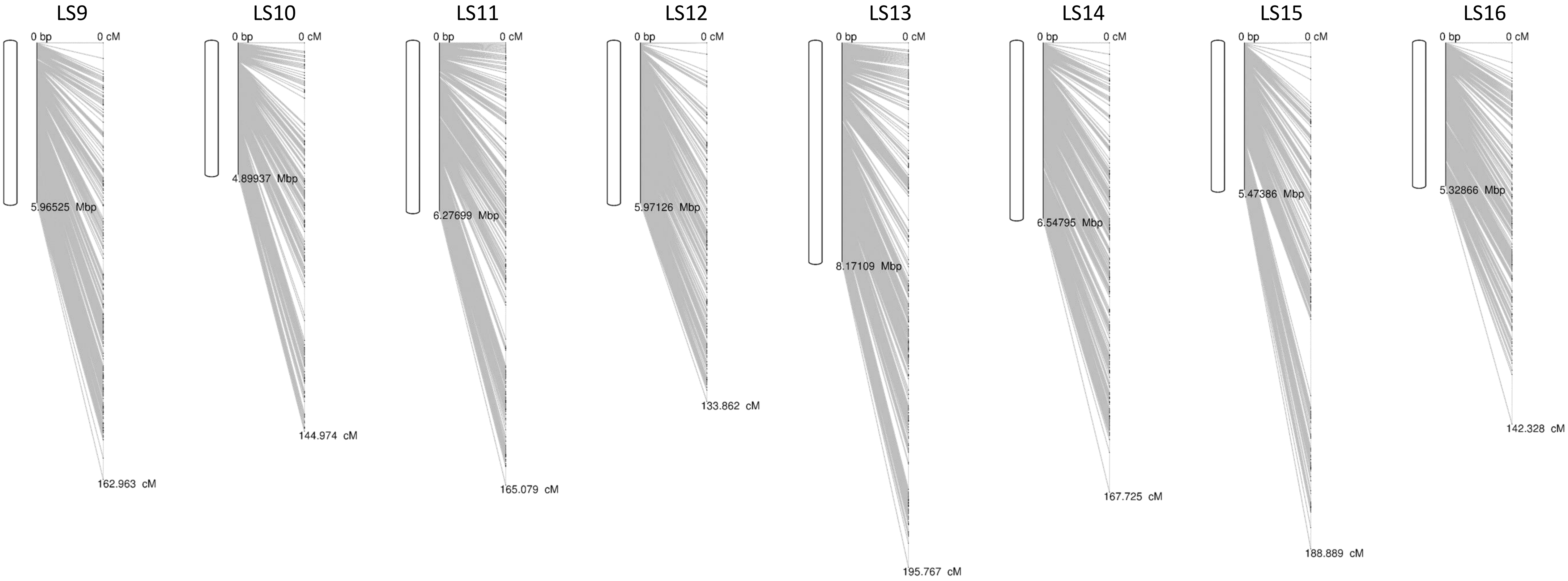

Supplementary Fig. 8 Linkage Map of SELDLA-extended *T. stictonotus*

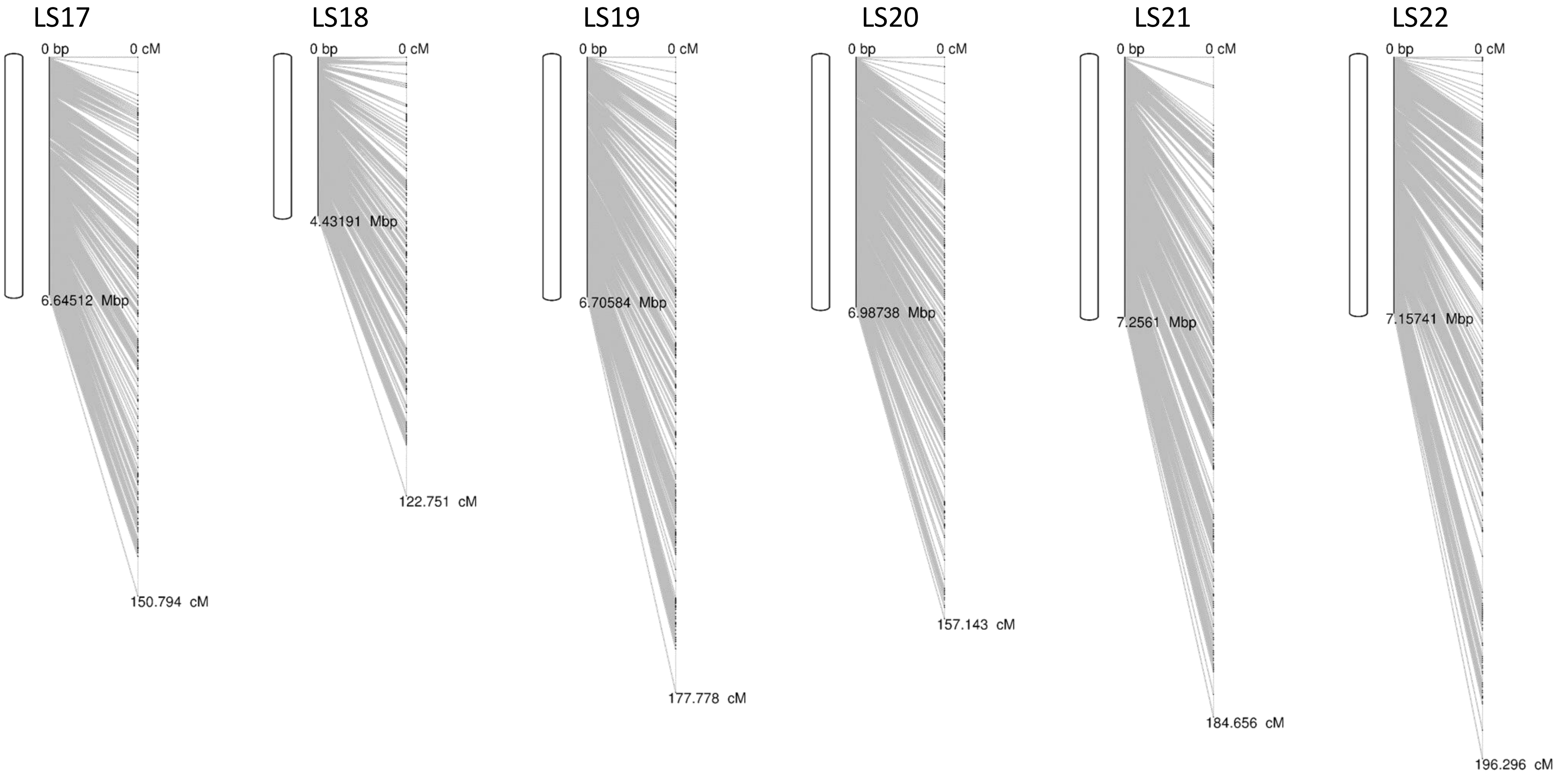

Supplementary Fig. 8 Linkage Map of SELDLA-extended *T. stictonotus*
